# Editorial: Ethnopharmacological Studies for the Development of Drugs With Special Reference to Asteraceae

**DOI:** 10.3389/fphar.2019.00955

**Published:** 2019-09-03

**Authors:** Sujogya Kumar Panda, Luis Cláudio Nascimento da Silva, Dinkar Sahal, Marco Leonti

**Affiliations:** ^1^Department of Biology, Katholieke Universiteit Leuven, Leuven, Belgium; ^2^Programa de Pós-Graduação da Universidade Ceuma, São Luís, Brazil; ^3^International Centre for Genetic Engineering and Biotechnology, New Delhi, India; ^4^Department of Biomedical Sciences, University of Cagliari, Cagliari, Italy

**Keywords:** Asteraceae, drug discovery, phytochemistry, anti-apoptosis, anti-diabetic, anti-inflammatory, antiparasitic, wound healing property

The Asteraceae (Compositae), also known as the daisy family, sunflower family, or thistle family, is one of the largest plant families with 32,913 accepted species divided into 1,911 genera and 13 subfamilies. The importance of the Asteraceae in traditional medicine and for food purposes is reported since antiquity and from different parts of the world. Despite the discovery of several secondary metabolites in Asteraceae, they attracted disproportionately little attention in the context of ethnopharmacological research, resulting in few systematic explorations and few commercialized products. The focus of this research topic is to make a special contribution to the ethnopharmacology of Asteraceae, considering their phytochemistry (as it relates to medical uses) and molecular pharmacology of their secondary metabolites. This special issue consists of five articles covering diverse topics related to the pharmacology of Asteraceae species.

One of the most important genera of the Asteraceae is probably *Artemisia*, as it is used worldwide in traditional medicine, as a source for spices (e.g., *Artemisia dracunculus*), ingredient for liquors (e.g., *Artemisia absinthium*), and as a source for artemisinin. The discovery of artemisinin fundamentally changed the treatment of malaria around the globe and led to the awarding of the Nobel Prize in Physiology or Medicine 2015 (Chen, 2016).

With advancements in mass spectrometry, Olennikov et al. studied 12 Siberian *Artemisia* species identifying 112 individual phenolic compounds through liquid chromatography and mass spectrometry analysis. Interestingly, the chemical profile appears to be very different between the analyzed species including rare plant phenolics, such as coumarin-hemiterpene ethers (lacarol derivatives) from *A. latifolia* and *A. tanacetifolia*; melilotoside from *A. tanacetifolia*; dihydrochalcones (davidigenin analogs) from *A. palustris*; chrysoeriol glucosides from *A. anethifolia*, *A. sericea* and *A. umbrosa*; eriodictyol glycosides from *A. messerschmidtiana*; and also some uncommon flavones and flavonols. Several of these *Artemisia* spp. are used in Siberia for the treatment of diabetes (Shah, 2014), and, therefore, a phytochemical and pharmacologic assessment was expected to provide interesting insights. In a series of *in vitro* assays, the *Artemisia* extracts showed inhibitory activity against principal enzymes of carbohydrate metabolism, such as α-amylase and α-glucosidase. The results obtained by Olennikov et al. reveal that Siberian Artemisia species have the potential to be useful in the development of alternative drugs for controlling diabetes.

In a very elegant research, Fattori et al. assessed the antinociceptive and anti-inflammatory potential of budlein A, a sesquiterpene lactone obtained from *Viguiera robusta* in a model of acute gout arthritis in mice. Gout was induced by intra-articular injection of monosodium urate (100 μg/10 μl). Animals pretreated with budlein A (1 or 10 mg/kg; 30 min prior the inflammation induction) showed lower levels of knee joint edema and mechanical hypersensitivity than the control group. These effects were shown to be associated with reduced recruitment of neutrophils and consequently reduced phagocytosis of monosodium urate crystals by neutrophils. The pretreatment with budlein A also lowered the transcription of expression of interleukin (IL) 1β and tumor necrosis factor-α genes in the knee joint. The results suggested the inhibition of nuclear factor-κB activation and impairment of the inflammasome assembly. Taken together, these findings suggest that budlein A might be used as a lead molecule for the treatment of certain inflammatory conditions.

Several plants from Asteraceae are used to treat infection with parasites, but the active compounds are not well studied (Panda and Luyten, 2018). A review article by Moraes Neto et al. discusses the aptitude of several Asteraceae plants for the development of drugs and pharmaceutical formulations against leishmaniasis and Chagas disease, two neglected tropical diseases. Several Asteraceae-derived compounds are highlighted based on their high selective activity against these protozoan parasites. Artemisinin and its derivatives, which showed activity against *Leishmania donovani*, *L. infantum*, and *L. major*, have attracted scientists from nanoscience, as nanoliposomal artemisinin proved as effective in *in vivo* mice model (Want et al., 2017). Psilostachyin (sesquiterpene from *Ambrosia tenuifolia*) is another interesting compound that showed *in vivo* efficacy against *L. mexicana* and *T. cruzi*. Furthermore, the sesquiterpene lactone deoxymikanolide obtained from *Mikania micrantha* showed *in vivo* activity against *T. cruzi*. It is also important to emphasize that the essential oils obtained from Asteraceae species are sources of bioactive compounds against trypanosomatids, in particular those from the genus *Artemisia*.

The capacity to promote wound healing is another well-known effect of certain Asteraceae species, and many Asteraceae are used for the treatment of skin lesions by traditional communities and in herbal medicine worldwide. Carvalho et al. reviewed several Asteraceae species and their associated compounds with wound-healing properties. Silibinin [obtained from *Silybum marianum* (L.) Gaertn], for example, showed high efficacy in different models of skin pathologies, such as toxic effects caused by nitrogen mustard in the mouse skin as well as experimental wounds. Other exciting results were seen in clinical trials focusing on complicated wounds including venous leg ulcers and foot ulcers of diabetic patients (Carvalho et al.) including *Ageratina pichinchensis* (Kunth) R.M. King and H. Rob. and *Calendula officinalis* L. Overall, the review by Carvalho et al. suggests that Asteraceae represent important sources for wound-healing compounds.

Costunolide (CST), a sesquiterpene lactone obtained from *Vladimiria souliei* (Franch.) Ling, is known to exhibit anti-inflammatory, anti-viral, and anti-tumor activities (Peng et al., 2017), while its potentially beneficial effects on liver injury are poorly understood. Mao et al. therefore, investigated the hepatoprotective effects of CST against lipopolysaccharide and D-galactosamine-induced acute liver injury in mice. The authors observed that CST (40 mg/kg) could significantly improve the pathological changes of hepatic tissue and that it reduced the lipopolysaccharide and D-galactosamine-induced increment of alanine aminotransferase and aspartate aminotransferase activities in the serum. Further data indicated that CST significantly reduces formation of malondialdehyde and reactive oxygen species and that it increased the activity of antioxidant enzymes in the hepatic tissues. Western blot data revealed that CST triggers the anti-oxidative defense system by inhibiting kelch-like ECH-associated protein 1 and nuclear factor-related factor 2 (in the cytosol and the nucleus), heme oxygenase-1 and nicotinamide adenine dinucleotide phosphate quinone oxidoreductase 1 activity. Moreover, CST led to a significant decrease in the protein expression of pro-inflammatory cytokines including IL 1β, IL 6, and tumor necrosis factor. The authors conclude that CST may be a potential therapeutic agent for attenuating acute liver injury.

The present research topic thus includes interdisciplinary research work expanding the knowledge about traditional uses of Asteraceae fostering the scientific field of ethnopharmacology globally. This research topic successfully gathered comprehensive information in the field of drug discovery related to diabetes, cancer, and human parasitosis. Furthermore, several bioactive compounds were characterized with regard to their molecular mechanisms related to wound healing and inflammation at large. We hope that this collection will inspire scientists from different fields of research focusing on the assessment of traditional medicine, derived from Asteraceae or other plant families, in the search for new pharmacological strategies.

## Author Contributions

All authors listed have made a substantial, direct, and intellectual contribution to the work and approved it for publication.

## Conflict of Interest Statement

The authors declare that the research was conducted in the absence of any commercial or financial relationships that could be construed as a potential conflict of interest.
